# An emerging framework for digital mental health design with Indigenous young people: a scoping review of the involvement of Indigenous young people in the design and evaluation of digital mental health interventions

**DOI:** 10.1186/s13643-023-02262-w

**Published:** 2023-07-01

**Authors:** Josie Povey, Buaphrao Raphiphatthana, Michelle Torok, Tricia Nagel, Patj Patj Janama Robert Mills, Joshua Russell Howard Sells, Fiona Shand, Michelle Sweet, Anne Lowell, Kylie Dingwall

**Affiliations:** 1 Menzies School of Health Research, Charles Darwin University, Casuarina Campus, Ellengowan Drive, Casuarina, NT 0810 Australia; 2 Black Dog Institute, University of New South Wales, Sydney, NSW 2052 Australia; 3 Northern Institute, Charles Darwin University, Casuarina Campus, Casuarina, NT 0810 Australia; 4 Menzies School of Health Research, Charles Darwin University, 10 Grevillia Drive, Alice Springs Campus, NT Australia

**Keywords:** Digital mental health, Indigenous, Adolescent, Young adult, Scoping review, Participatory, Efficacy, Co-design

## Abstract

**Background:**

Indigenous young people worldwide possess unique protective factors that support wellbeing. However, they experience mental illness at higher rates than their non-indigenous counterparts. Digital mental health (dMH) resources can increase access to structured, timely, and culturally tailored mental health interventions by reducing structural and attitudinal barriers to accessing treatment. The involvement of Indigenous young people in dMH resource development is recommended, however, no guidelines exist on how this can best be facilitated.

**Methods:**

A scoping review examining processes to involve Indigenous young people in developing or evaluating dMH interventions was conducted. Studies reported between 1990 and 2023 involving Indigenous young people aged 12–24 years, originating from Canada, the USA, New Zealand, and Australia, in the development or evaluation of dMH interventions were eligible for inclusion. Following a three-step search process, four electronic databases were searched. Data were extracted, synthesized, and described under three categories: dMH intervention attributes, study design, and alignment with research best practice. Best practice recommendations for Indigenous research and participatory design principles derived from the literature were identified and synthesised. Included studies were assessed against these recommendations. Consultation with two Senior Indigenous Research Officers ensured Indigenous worldviews informed analysis.

**Results:**

Twenty-four studies describing eleven dMH interventions met inclusion criteria. Studies included formative, design, pilot, and efficacy studies. Overall, most included studies demonstrated a high degree of Indigenous governance, capacity building, and community benefit. All studies adapted their research processes to ensure that local community protocols were followed and most aligned these within an Indigenous research paradigm. Formal agreements regarding existing and created intellectual property and implementation evaluations were rare. Outcomes were the primary focus of reporting, with limited detailed descriptions of governance and decision-making processes or strategies for managing predictable tensions between co-design stakeholders.

**Conclusions:**

This study identified recommendations for undertaking participatory design with Indigenous young people and evaluated the current literature against these criteria. Common gaps were evident in the reporting of study processes. Consistent, in-depth reporting is needed to allow assessment of approaches for this hard-to-reach population. An emergent framework, informed by our findings, for guiding the involvement of Indigenous young people in the design and evaluation of dMH tools is presented.

**Trial registration:**

Available via osf.io/2nkc6

**Supplementary Information:**

The online version contains supplementary material available at 10.1186/s13643-023-02262-w.

## Background

Indigenous young people worldwide experience unique facilitators of resilience, including strong connections with family, community, spirituality, land, and lore, which promote wellbeing [1–4]. Despite this, they experience higher rates of mental illness, substance misuse, and suicide than their non-Indigenous counterparts [5, 6]. Globally, in high-income countries, rates of suicide for Indigenous people are highest among adolescent and rural and remote living populations [7], providing unique challenges in the delivery of services [8, 9]. Mental health services often fail to be accessible and culturally safe for Indigenous people [10], and the development and evaluation of culturally tailored interventions is required [11–13]. Digital mental health (dMH) tools can address service delivery gaps by reaching marginalised and geographically isolated young people [14].dMH services use a digital platform (e.g. smartphone, website) to deliver mental health services [15]. They rely on computerised systems that are less resource-dependent and easily scalable, thereby reducing cost, increasing accessibility, and improving treatment fidelity [16, 17]. Participatory design in dMH resource development is widely recommended and may improve acceptability and uptake [18]. Participatory design involves end-users in generative, playful, and experiential activities throughout the design and evaluation of dMH resources [19]. Several challenges in engaging children and young people in participatory design have been reported. These include diverse user dMH design preferences, young people's frustration with the pace and scope of projects, and the need to communicate scope and parameters while still engaging young people in fun, interactive, and age-appropriate activities [20, 21]. To ensure success, participatory design requires consideration of several principles, including acknowledging diversity [22, 23], shared decision-making [17, 19, 24], participant and research team upskilling, and strategies to manage the predictable tensions which arise within participant and stakeholder groups [20, 21, 25–28]. Establishing criteria for detailed reporting of processes and outcomes will assist participatory design in becoming a more methodologically sound approach [20, 29, 30]. Through our review of the relevant literature reporting participatory design, we have previously identified and reported several consistent themes [31]. We summarise these below in Table 1.

**Table 1:** Principles of participatory design derived from the literature

1. Engage throughout in an iterative process of design, development, and review
2. Acknowledge youth diversity and avoid a one size fits all approach
3. Generate resources through experiential, playful action-based activities
4. Respect/upskill/empower young people (users)
5. Shared decision-making throughout to effectively reflect young people’s views
6. Address tensions between user preferences and experts
7. Manage expectations in accordance with resource availability
8. Evaluate process through user perspectives
9. Report process as well as outcome

Privileging Indigenous young people’s voices by engaging them in the development and evaluation of dMH solutions through participatory methods is essential for developing relevant, user-friendly, and engaging dMH tools [20, 32–38]. Meaningful engagement enables better research practices and improves the likelihood of producing more acceptable, culturally responsive tools to address the current unmet need of this hard-to-reach population [22]. This user-centered design approach aligns with the recommendations from the World Health Organization and best practice Indigenous research principles [36, 39–43].

Several guidelines originating from Australia [40, 44], New Zealand [39, 42], and Canada [41] outline best practice approaches for engaging Indigenous people in research. The Aboriginal and Torres Strait Islander Quality Appraisal Tool (Aboriginal and Torres Strait Islander QAT) was developed based on these guidelines to increase the quality and transparency of Aboriginal and Torres Strait Islander research practice and reporting [45]. This 14-item tool, to our knowledge, it is the only Indigenous research quality assessment tool available globally [45]. Consequently, this tool is used to assess the quality of studies included in this scoping review regarding Indigenous control and governance, preservation of cultural and intellectual property, capacity strengthening, and benefit to individuals and the community. Critically reviewing research practices using such tools in the design and development of dMH resources involving Indigenous young people ensures ethical guidelines are upheld, to safeguard and inform best practice into the future [37].

Although participatory design is commonly recommended, there is limited in-depth reporting of the processes used to design and evaluate resources. This gap in the literature limits progress in determining the link between dMH co-design, uptake, and effectiveness [20, 46, 47] and recommendations for involving Indigenous young people in dMH development or evaluation do not currently exist. Two prior systematic reviews have described dMH products and outcomes for Indigenous young people [35, 48] but previous reviews have not examined the processes undertaken to engage Indigenous young people in developing or evaluating dMH tools. Therefore, this scoping study aims to review and synthesise research involving Indigenous young people in developing or evaluating dMH interventions to describe the methods used and to assess the alignment of these methods with best practice recommendations for Indigenous health research and participatory design.

## Methods

The study protocol for this scoping review has been published previously [49]. It follows guidelines proposed by Arksey and O'Malley [50] and the subsequent modifications proposed by Levac et al. [51] and Peters et al. [52] and involves a six-stage process: (1) identifying research question; (2) identifying relevant studies; (3) study selection and data extraction, with methods refined using an iterative process throughout [51]; (4) data charting; (5) collating, summarising and reporting results. Additionally, step 6 consultation engages two senior Indigenous researchers throughout scoping review processes to ensure analysis and findings are informed by Indigenous worldviews [53]. To ensure thorough and transparent reporting, we adhered to the Preferred Reporting Items for Systematic Reviews and Meta-Analysis 2020 (PRISMA2020) statement [53, 54]. The PRISMA 2020 checklist is included as Supplementary file 1.

### Information sources

A systematic search was conducted, utilising the following databases: EBSCOhost databases (Academic Search Premiere, Computer, and Applied Science Complete, CINAHL Plus with Full text, MEDLINE with full text, APA PsychArticles, Psychology, and Behavioural sciences collection, APA PsychInfo); PubMed; Scopus; Informit and Google (limited to the first 200 results). Informit and Google were included to capture grey literature or unpublished studies. Reference lists of potential studies and reviews were examined for additional studies. Identified dMH tools and facilitating university websites were searched for further information. Where processes were not adequately described, additional information was requested from corresponding authors via email.

### Eligibility criteria

Research studies of any design (excluding reviews), reported in English from January 1st, 1990 to March 3rd 2023, which developed, evaluated, or tested dMH approaches with Indigenous young people were eligible. This timeframe was chosen due to its alignment with the emergence of dMH approaches [54, 55]. Eligible studies involved Indigenous young people originating from Australia (Aboriginal and Torres Strait Islander), New Zealand (Māori), Canada (Inuit, First Nations people), and the United States of America (First Nations people). These countries were chosen as Indigenous people in these developed first-world countries have similar experiences of colonisation, persistent health inequities and can often reside in remote and rural areas. The original protocol was updated to allow an additional study to be included. This required an update of the criterion related to the percentage of the sample identifying as an Indigenous young person from 50 to 49% (see Table 2). For the purpose of this review, ‘young people’ refers to those aged 10–24 years, representing a broad definition of adolescence [56].

**Table 2:** Inclusion and Exclusion criteria

**Inclusion criteria**
• Minimum 49% of study participants identify as Indigenous
• Minimum 50% of study participants are aged 10–24 years
• Studies based in Australia, Canada, New Zealand, United States of America
• Interventions targeting the mental health of young people (including health promotion/psychoeducation, prevention/early intervention, crisis intervention/suicide prevention, treatment, recovery, and mutual/peer support)
• Young people are involved in dMH design, development, or evaluation
• Interventions delivered using Information Communication Technology (smartphone, iPad, websites, computers, and other digital devices)
• The primary focus of the study is mental health problems or well-being outcomes, including suicidality, substance use, and smoking
**Exclusion criteria**
• Not related to mental health/wellbeing (i.e. physical health as outcome)
• Study population outside of the above culture, age, and geographic parameters
• Young people are not involved in the design or evaluation or are not the intended target audience of the dMH intervention
• Non-English language studies (due to limitations in time/resources)
• Studies focused on telepsychiatry via videoconferencing or telephone; without a significant engagement with apps, websites, email, or other computerised systems
• Electronic health or medical records, decision support tools for clinicians, analytic services, services that primarily provide support and education to health professionals, clinical practice management software, and clinical workflow and communication software

Studies reporting on the design, development, or evaluation of mental health interventions, which use a digital platform (e.g. smartphone, tablet device, website, wearable devices) to deliver mental health services (e.g. health promotion/psychoeducation, prevention/early intervention, crisis intervention/suicide prevention, treatment, recovery and mutual/peer support) were eligible. Studies describing interventions such as telepsychiatry and video psychiatry without the use of other computerised methods (e.g. websites, online game, or SMS support) were excluded, as these services are more closely aligned with face-to-face service delivery models [16, 17]. Studies with the primary treatment goal of improving mental health or wellbeing (i.e., psychological distress, anxiety/stress management, suicidality, substance use, and smoking) were included. Studies with a physical health focus (e.g. diabetes, HIV management) were excluded. Electronic health or medical records, decision or education support tools for health professionals, analytic services, clinical practice management software, and clinical workflow and communication software were also excluded [15]. A complete list of inclusion and exclusion criteria is shown in Table 2.

### Search strategy

A three-step search process was used, which followed recommendations outlined by Joanna Briggs Institute [57]. An initial limited search of two databases (EBSCOhost and PubMed) was undertaken independently by two authors (JP, BR). Titles, abstracts, and keywords of retrieved articles were reviewed to find additional search terms before three reviewers (JP, BR, MT) met to finalise keywords. The final keywords for each database are included in Supplementary file 2. Two independent reviewers (JP, BR) used the updated search terms to conduct a second search across all databases, including grey literature databases. Searches were conducted on September 18th, 2020, and updated on February 22nd, 2022 and March 3rd 2023. Lastly, reference lists of potential studies and reviews were examined for additional studies.

### Study selection

PRISMA-Scr guidelines were followed for the selection of studies. Citations and abstracts were exported to Endnote referencing system, duplicates removed and then remaining citations were exported to excel for screening. Following the initial search on September 2020, two reviewers (JP, BR) independently reviewed a random 10% (*n* = 150) of retrieved records by article titles and abstracts and applied inclusion and exclusion criteria (Table 2). Reviewers met to discuss abstract screening and selection of articles. An initial inter-rating agreement kappa result of 0.66 (94.6% agreement) was reached. Following discussion, changes were made to the inclusion criteria. A second agreement kappa of an additional random 10% (*n* = 153) of identified articles achieved a result of 0.81 (98.0%: ‘almost perfect’ to ‘perfect’ agreement). Full-text articles were retrieved by the first author (JP) following full list screening at the title and abstract stage. Full-text articles were reviewed by the same two reviewers independently for eligibility. A 10% full-text screening (*n* = 13) inter-rating agreement kappa revealed 100% agreement. For subsequent searches, title and abstract screening and full-text review for eligibility was completed by JP only. The first author prepared the final list of included articles. If the first author was unsure of a study’s eligibility throughout full-text screening, three reviewers (JP, BR, MT) reviewed the articles in question before discussion to reach a consensus. Decisions for inclusion or exclusion were recorded.

### Data extraction and synthesis

Data extracted from each full-text article were classified under three broad categories: (1) dMH intervention attributes, (2) study design, and (3) alignment with research best practice, and synthesised in table format using Microsoft excel. Data extraction variables, outlined in Supplementary file 3, were based on the Aboriginal and Torres Strait Islander QAT items and our identified principles of participatory design [40, 45, 58]. Data extraction variables were refined in response to the emerging findings, as often occurs throughout the iterative process of scoping reviews [57]. Data extraction forms were independently tested by two reviewers (JP, BR) on a random sample of two (13%) studies to ensure accuracy, consistency, and validity of captured information [51, 57]. The first author then extracted data from the remaining included articles using Microsoft excel. Initially qualitative content analysis [59] was undertaken to determine reported activities related to each data extraction variable. Key elements within each data extraction variable identified through content analysis were then discussed within the research team to further develop criteria for applying consistent ratings for the Aboriginal and Torres Strait Islander QAT and our identified principles of participatory design. On three occasions, preliminary findings were documented and used to create discussion within consultation meetings with two Senior Indigenous Research Officers (PPJRM, JRHS). Written notes were taken during consultation meetings as per recommended best practice [51]. The research team further revised and refined themes through a series of meetings. All search results and full-text articles were managed through Endnote X9, using groups, annotations, and notes features.

## Results

**Fig. 1:**
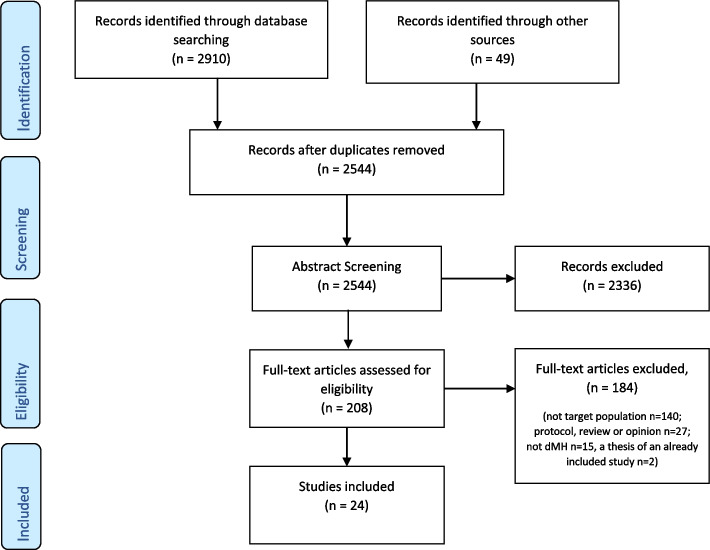
PRISMA diagram

Database searches conducted on September 18th, 2020, revealed 1806 records, with an additional 23 located through other sources. We updated the search on February 22nd, 2022 and March 3rd, 2023, to include articles published between September 2020 and March 2023, which identified a further 1104 records. A further 26 were identified primarily through a hand search of eligible studies (2959 total). Following the removal of duplicates (415 records), 2544 remained, which were then screened for relevance at the title and abstract level. After excluding 2336 articles not meeting inclusion criteria, 208 full-text articles were reviewed. A further 184 articles were excluded for reasons as follows: not meeting criteria for the target population (*n* = 140), a review or opinion piece (*n* = 27), not including a digital mental health intervention (*n* = 15), thesis of an already included study (*n* = 2). The 24 studies identified for inclusion were published between 2010 and 2023 (Fig. 1). The studies originated from the USA (*n* = 8), New Zealand (*n* = 6), Australia (*n* = 7), and Canada (*n* = 3).

Grey literature sources, including websites, theses, and study protocols, were not included in the final list but were used to supplement understanding. As the information required for this review was inconsistently presented in the academic literature or often required further clarification, additional information was sought from all corresponding authors of included studies via email.

### Digital mental health interventions

The 24 studies described 11 unique dMH programs with varying purposes, delivery modes, and therapeutic basis (Table 3). Programs sought to address smoking (*n* = 1), depression (*n* = 2), suicide prevention (*n* = 2), hazardous drinking (*n* = 4), and wellbeing support (*n* = 2). Almost all included cultural adaptions, most commonly through graphic design. Some incorporated written or audio Indigenous language [60, 61] or full gender-matched audio (in English) to overcome literacy challenges and follow cultural protocols [62]. On one occasion, the research team determined that designing the dMH resource specifically to suit one cultural group could further stigmatise that group; therefore, on advice from senior cultural informants chose not to culturally adapt the resource [63]. One resource, SPARX, was designed universally for all New Zealand young people; however, it incorporated Māori designs from the outset to ensure appeal to a wide variety of young people [61, 64–67]. A further body of work explored Inuit young people’s perceptions of the cultural appropriateness of the Māori designed SPARX intervention through qualitative and piloting processes, and then adapted the resource to suit the context [68–70].

**Table 3:** Digital mental health resources described in included

Clinical Focus	Name	Clinical aim	Delivery mode	Target population	Therapeutic basis	Intervention strategy	Cultural design/adaptation
Smoking, alcohol, and other drugs	SmokingZine [71, 72]	Smoking cessation and prevention	Website	American Indian/American Native youth	NR	Psychoeducation	Yes
	e-SIB [63]	Reducing hazardous drinking (alcohol)	Website	New Zealand Youth	NR	Screening and brief intervention	No
	Youth CHAT [60]	Screening (AOD, sexual and mental health)	Tablet device	New Zealand Youth	NR	Screening and brief intervention	Yes
	CHAT [73]	Alcohol exposed pregnancy prevention	Web-based	American Indian/American Native teens	Motivational interviewing	Motivational interviewing and psychoeducation	Yes
	BRAVE [74–76]	Alcohol and violence	SMS (Video content)	American Indian/American Native Youth	NR	Psychoeducation and brief intervention	Yes
Depression	SPARX [61, 65–68]	Depression (mild to moderate)	Web-based	New Zealand Youth	CBT	Gamified CBT	Yes
	I-SPARX [68–70]	Depression (mild to moderate)	Web-based	Inuit Youth	CBT	Gamified CBT	Yes
Suicide prevention	Project Life [77]	Suicide prevention	Website	Alaskan Native Youth	CBT	Digital storytelling	Yes
	Ibobbly [78–80]	Suicide prevention	Smartphone App	Aboriginal and Torres Strait Islander Youth (16–35 years)	CBT	Psychoeducation and brief intervention	Yes
Wellbeing support	Stayin’ OnTrack [81]	Fathering and mental health support	Website and SMS	Young Aboriginal Fathers	NR	Digital Storytelling Mood tracking Brief intervention	Yes
	AIMhi-Y [31, 58]	Early intervention wellbeing	Smartphone App	Aboriginal and Torres Strait Islander Youth (10–18 years)	Low intensity-CBT	Gamified brief intervention	Yes

*NR* not reported

### Study methodology, methods, and participant demographics

Overall, formative, design, and feasibility studies using primarily qualitative methods (14/24; 58%) were more common than pilot or efficacy studies which used mainly quantitative methods (9/24; 41%) (Tables 4 and 5). The authors determined the study stage based on the study description and definitions derived from the literature [82–84], as terms and definitions were used inconsistently between studies. The two dMH tools reported over the most stages were SPARX and BRAVE. No studies reported on implementation.

**Table 4:** Study stage definitions

Formative	Studies focused on young people’s experiences of mental health and wellbeing and explored the acceptability of dMH tools to address an identified need
Design	Studies focused on designing or developing a resource, which could include identifying preferable features
Feasibility	Studies aimed at working toward the adaptation or refinement of a dMH intervention. This includes studies describing acceptability, design, or prototype testing without a focus on determining a treatment effect
Pilot	Studies conducted before an efficacy or effectiveness study which resemble the planned study or part of the planned study but on a smaller scale
Efficacy	Studies testing the efficacy or effectiveness of the dMH tool in research settings with research therapists/providers or community settings with community therapist providers
Implementation	Studies assessing or describing large-scale implementation at a population level. Studies often test the dMH tool in 'real world' settings

**Table 5:** Study design, methods, and sample by dMH resource

Name	Stages reported	Study design	Sample				
			Youth (*n*)	Age range (years)	Other^a^ (*n*)	Female (%)	Female (%)
Smoking Zine	Design/feasibility [71]	Qualitative (focus groups)	12	13–18	0	58	100
	Pilot [72]	Two-arm randomised pilot	113	13–26	0	61	84
SPARX	Formative [85]	Qualitative (focus groups)	39	13–16	0	26	49
	Design [61]	Qualitative (culturally informed focus groups, surveys)	19	16–18	7	–^b^	100
	Feasibility [65]	Qualitative (interviews)	6	14–16	0	83	100
	Efficacy [66]	Stepwise cohort study	40	14–17	0	0	67
I-SPARX	Formative [68]	Qualitative (interviews, focus groups)	11	13–18	7	–	100
	Formative [69]	Qualitative (online interviews)	9	16–22	0	33	100
	Pilot [70]	Modified randomised control study	24	13–18	0	–	100
e-SIB	Efficacy [63]	Two arm RCT (online)	1789	1724	0	66	100
Project Life	Feasibility [77]	Qualitative (interviews, survey)	299	9–17	0	–	100
Youth CHAT	Feasibility [60]	Community-based participatory research (focus group, interviews)	30	< 25	2	93	90
ibobbly	Design [80]	Qualitative (interviews, focus groups)	–	–	–	–	–
	Pilot [78, 79]	Two arm RCT (app usage data)	61	18–56	0	64	94
Stayin’ OnTrack	Design/feasibility [81]	Participatory design (culturally informed focus groups)	20	18–25	0	0	100
CHAT	Design/feasibility [73]	Qualitative (interviews, focus groups)	15	15–19	15	100	100
AIMhi-Y	Formative/design [58]	Participatory design (co-design workshops, online survey)	45	10–18	0	47	100
	Design [31]	Participatory design (co-design workshops, interviews, reference group)	65	8–18	6	53	100
	Pilot [86]	Mixed methods (non-randomised pre-post, outcomes measures, interviews)	30	12–18	0	43	100
BRAVE	Formative/design/feasibility [75]	Participatory research (interviews, surveys)	30	18–24	8	0	100
	Efficacy [74, 76, 87]	Two arm crossover RCT (user engagement data)	833	15–24	0	66	100

^a^Health professionals, family, or key informants

^b^Missing data

Data collection primarily occurred face to face [61, 62, 68, 77, 85] and online [58, 63] with varying degrees of peer, service provider, or researcher support [58, 66, 70, 72]. Two studies reported on user engagement data throughout efficacy testing [76, 79]. Two studies undertook exclusively online recruitment via existing networks using email [63] and social media [88]. They both delivered interventions remotely with automated data collection. Both maintained a large sample over an extended timeframe (*n* = 1415 (79% of original recruitment) over five months [63] and *n* = 833 (79% of original recruitment) over 9 months [88]). Study settings included education [58, 63, 65, 77, 85], health [60, 73, 81], community [66, 68–72, 75, 78, 87], and juvenile justice [67].

### Alignment with best practice in Indigenous research and principles of participatory design

To assess best practice processes within the scoped literature, the first author (JP) initially assessed each study against the Aboriginal and Torres Strait Islander QAT [45] and principles of best practice in participatory design derived from the literature presented earlier in Table 1 (see results in Tables 6 and 7). Despite being developed in Australia, the Aboriginal and Torres Strait Islander QAT [45] aligns with principles of Indigenous research within the international context, thus has been used to assess international studies. Studies were given ratings of 'yes', 'partial', ‘no’ or ‘unclear’, based on the information available. Many of the criteria were subjective, and ratings were not always clear from the information presented. All corresponding authors were contacted for further information, with most (72%) responding to clarify additional processes not outlined in the academic literature. Where there were discrepancies or where processes remained unclear, discussions occurred within the research team to confirm ratings. All Aboriginal and Torres Strait Islander QAT [45] ratings were discussed with the Indigenous research team members (PPJRM, JRHS) to ensure findings were assessed from an Indigenous perspective.

**Table 6:** Studies rated using the Aboriginal and Islander QAT Tool

	**Indigenous governance**				**Respect for cultural and intellectual property**				**Capacity building**			**Beneficial outcomes**			**Overall assessment**
	Q1	Q2	Q3	Q4	Q5	Q6	Q7	Q8	Q9	Q10	Q11	Q12	Q13	Q14	
**Smoking Zine **[71, 72]															
Taualii et al. 2010	P	P	U	P	P	U	U	U	U	P	P	U	U	U	Collaborative project with health and representative bodies but unclear who led and what governance processes were in place, some adaption of research processes to suit local need evident
Bowen et al. 2012	P	P	U	P	P	U	U	U	U	U	U	U	U	U	
**SPARX **[61, 65–68]															
Shepherd et al. 2015	Y	Y	Y	Y	Y	U	U	P	Y	Y	Y	Y	Y	Y	An extensive program of work, with ongoing implementation, Indigenous leadership and governance of research processes evident, cultural paradigm and adaption to local need evident
Shepherd et al. 2018	Y	Y	Y	Y	Y	U	U	P	Y	Y	Y	Y	Y	Y	
Fleming et al. 2019	Y	Y	Y	Y	Y	U	U	U	Y	U	Y	Y	Y	Y	
Fleming et al. 2012	Y	Y	Y	Y	Y	U	U	U	Y	U	Y	Y	Y	Y	
**I-SPARX**															
Litwin et al. 2023	Y	Y	Y	Y	Y	U	P	P	Y	Y	P	P	Y	Y	Inuit governance and capacity strengthening evident, ongoing work with initial benefit demonstrated, adaption to local need evident
Thomas et al. 2022	Y	Y	Y	Y	Y	U	P	P	Y	Y	P	P	Y	Y	
Bohr et al. 2023	Y	Y	Y	Y	Y	U	P	P	Y	Y	P	P	Y	Y	
**e-SIB **[63]															
Kypri et al. 2012	P	Y	Y	Y	Y	U	U	U	Y	Y	Y	Y	Y	Y	Māori governance and capacity-strengthening evident, benefit, and translation of findings into practice demonstrated
**Project life** [77]															
Wexler et al. 2013	Y	Y	Y	Y	Y	U	P	P	Y	Y	U	P	Y	Y	The project was led by a Tribal Health Board, capacity strengthening and adaption of processes to suit local need
**Youth CHAT **[60]															
Goodyear-Smith et al. 2016	Y	Y	U	U	Y	U	U	U	Y	Y	Y	Y	P	Y	Adaption of processes to suit the local need, consultation, and engagement with the Māori community but unclear governance role or structure, benefit to community evident
**Ibobbly **[78–80]															
Tighe et al. 2017	Y	Y	U	P	Y	U	U	U	Y	Y	Y	Y	P	P	Adaptions of the process to suit the local need, comprehensive consultation prior to the research, benefit to individuals evident, some Indigenous governance, and leadership structure evident
Tighe et al. 2020	Y	Y	P	P	Y	U	U	U	Y	Y	Y	Y	P	P	
BDI, 2015	Y	Y	P	U	Y	U	U	U	Y	Y	Y	Y	Y	Y	
**Stayin' OnTrack **[81]															
Fletcher et al. 2017	Y	Y	Y	Y	Y	U	P	P	Y	Y	Y	Y	Y	Y	Strong focus on capability building and community governance, dissemination, and benefit to community evident
**CHAT **[73]															
Hanson et al. 2020	Y	Y	P	Y	Y	U	U	U	Y	P	U	U	Y	Y	Indigenous involvement and governance evident and adaptions of the process to suit local needs; Indigenous leadership unclear
**AIMhi-Y **[31, 58]															
Povey et al. 2020	Y	Y	Y	Y	Y	U	U	P	Y	Y	P	P	Y	Y	Adaptions of the process to suit the local need, Indigenous governance, leadership, and capability building evident, analysis informed by Indigenous youth and researchers
Povey et al. 2022	Y	Y	Y	Y	Y	U	U	P	Y	Y	P	P	Y	Y	
Dingwall et al. 2023	Y	Y	Y	Y	Y	U	U	P	Y	Y	Y	Y	Y	Y	
**BRAVE **[74, 75, 87]															
Stephens et al. 2020	Y	Y	Y	Y	Y	U	Y	Y	Y	Y	Y	Y	U	Y	The project was led by a Tribal health board, benefit was demonstrated, and processes for data collection and data sovereignty were governed by Indian Health Board
Rushing et al. 2021	Y	Y	Y	Y	Y	U	Y	Y	Y	Y	Y	Y	U	Y	
Rushing 2020/21	Y	Y	Y	Y	Y	U	Y	Y	Y	Y	Y	Y	P	Y	
Wrobel et al. 2022	Y	Y	Y	Y	Y	U	Y	Y	Y	Y	Y	Y	Y	Y	

*Y* = yes, *P* = partially, *U* = no explicit statements in the body of the text to provide evidence, *N* = articulated they did not do something or did something contrary to best practice

1. Did the research respond to a need or priority determined by the community?

2. Was community consultation and engagement appropriately inclusive?

3. Did the research have Indigenous^a^ research leadership?

4. Did the research have Indigenous governance?

5. Were local community protocols respected and followed?

6. Did the researchers negotiate agreements in regards to rights of access to existing Indigenous peoples' intellectual and cultural property?

7. Did the researchers negotiate agreements to protect Indigenous ownership of intellectual and cultural property created through the research?

8. Did Indigenous peoples and communities have control over the collection and management of research materials?

9. Was the research guided by an Indigenous research paradigm?

10. Does the research take a strengths-based approach, acknowledging and moving beyond practices that have harmed Indigenous peoples in the past?

11. Did the researchers plan to and translate the findings into sustainable changes in policy and/or practice?

12. Did the research benefit the participants and Indigenous communities?

13. Did the research demonstrate capacity strengthening for Indigenous individuals?

14. Did everyone involved in the research have opportunities to learn from each other?

^a^The Aboriginal and Torres Strait Islander Quality Appraisal Tool uses the term ‘Aboriginal and Torres Strait Islander’ throughout. We have replaced this term with Indigenous to suit this scoping review study which reviews studies within an international context. Limitations of this approach are highlighted in the limitations section

**Table 7:** Studies rated using recommended principles of participatory design derived from the literature

	**Engagement**			**Partnership**				**Evaluation and reporting**		**Overall assessment**
	Q1	Q2	Q3	Q4	Q5	Q6	Q7	Q8	Q9	
**SmokingZine**										
Taualii et al. 2010	Y	Y	Y	P	U	U	U	U	Y	Multiple stages were reported with a reasonable degree of detail of youth involvement, youth diversity acknowledged; unclear partnership criteria
Bowen et al. 2012	Y	Y	Y	P	U	U	U	U	Y	
**SPARX**										
Shepherd et al. 2015	Y	Y	Y	Y	U	U	U	Y	Y	Ongoing engagement of young people in design and evaluation evident over several studies, evaluation of co-design processes provided, acknowledgment of the diversity of young people evidenced by multiple studies with different groups (i.e., those in alternative education, Māori adolescents), the influence of young people in decision-making processes was difficult to establish
Shepherd et al. 2018	Y	Y	Y	Y	U	U	U	P	Y	
Fleming et al. 2019	Y	Y	Y	Y	U	U	U	P	Y	
Fleming et al. 2012	Y	Y	Y	Y	U	U	U	U	Y	
**I-SPARX**										
Litwin et al. 2023	Y	Y	Y	Y	P	U	U	Y	P	Several studies describing formative evaluation and piloting, wide range of communities and young people recruited, evaluation of process evident, young people’s decision-making influence, tensions arisen not discussed
Thomas et al. 2022	Y	Y	Y	Y	P	U	U	Y	P	
Bohr et al. 2023	Y	Y	Y	Y	P	U	U	U	Y	
**s-SIB**										
Kypri et al. 2012	Y	P	P	P	U	U	U	P	P	Several formative studies report the involvement of Māori young people throughout development, however unsure of their role, frequency of engagement, or influence in the process
**Project Life**										
Wexler et al. 2013	Y	Y	Y	Y	Y	U	U	Y	Y	Interviews and community forums provided feedback on co-design processes with respect, shared decision-making, and upskilling evident. Unlikely, tensions between stakeholders arose as individualised content was generated (i.e. each participant made their digital story)
**Youth CHAT**										
Goodyear-Smith et al. 2016	P	Y	Y	Y	U	U	U	U	Y	Minimal information available on the initial development process. Implementation was co-designed with young people; however, unsure of upskilling or decision-making processes
**ibobbly**										
Tighe et al. 2017	P	Y	Y	Y	U	U	U	U	P	Information on initial co-design processes and sample not available, some decision-making processes described during re-design and challenges such as resourcing and representativeness explored
Tighe et al. 2020	P	Y	Y	Y	U	U	U	U	P	
Black Dog Institute, 2015	Y	Y	Y	Y	P	U	U	U	Y	
**Stayin' on Track**										
Fletcher et al. 2017	Y	Y	Y	Y	Y	U	U	Y	Y	Evaluation of co-design processes evident in the form of community feedback; respect, shared decision-making, and upskilling evident
**CHAT**										
Hanson et al. 2020	Y	Y	Y	P	U	U	U	U	Y	A good description of formative work, unclear partnership criteria
**AIMhi-Y**										
Povey et al. 2020	Y	Y	Y	Y	U	U	U	U	Y	Detailed description of formative work including a large sample with diversity noted, decision-making processes detailed, and tensions between stakeholders presented to some degree. Key learnings discussed. Small pilot study with evaluation of process evident
Povey et al. 2022	Y	Y	Y	P	P	P	P	U	Y	
Dingwall et al. 2023	Y	Y	Y	Y	P	U	U	Y	Y	
**BRAVE**										
Stephens et al. 2020	Y	Y	Y	Y	P	U	U	U	Y	In-depth account of formative and design work, engagement, and partnerships were evident. Multiple stakeholders were involved throughout development, decision-making processes and how tensions were managed were not detailed
Rushing et al. 2021	Y	Y	Y	Y	P	U	U	U	Y	
Rushing 2021	Y	Y	Y	Y	P	U	U	U	Y	
Wrobel et al. 2022	Y	Y	Y	Y	P	U	U	U	Y	

*Y* = yes, *P* = partially, *U* = no explicit statements in the body of the text to provide evidence, *N* = articulated they did not do something or did something contrary to best practice

1. Did the research engage young people throughout an iterative design, development, and review process?

2. Did the research acknowledge youth diversity and avoid a one-size-fits-all approach?

3. Did the research generate resources through experiential, playful, or action-based activities?

4. Did the research respect, upskill, and empower young people while ensuring their safety?

5. Did the research describe a shared decision-making process to ensure young people's views were effectively reflected?

6. Did the research address the predictable tensions between user preferences, experts, and other stakeholders?

7. Did the research manage expectations in accordance with resource availability?

8. Did the research evaluate the process from the users' perspective?

9. Did the research adequately report their processes as well as the outcomes?

**Fig. 2:**
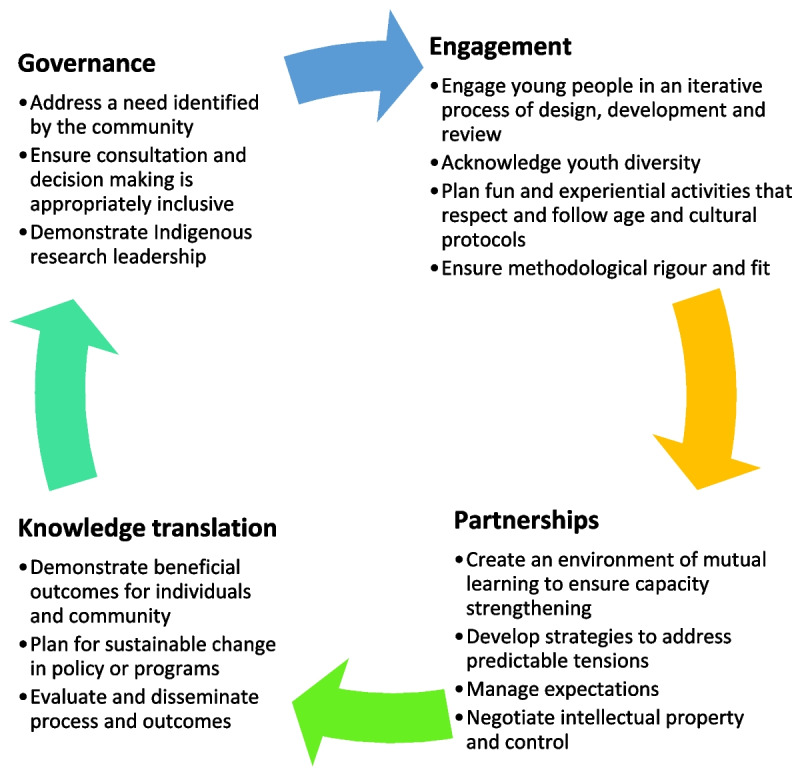
An emerging framework for the development of dMH resources with Indigenous young people

Following individual assessment of each study, against both criteria, we identified several commonalities between the items. The first author then reviewed and integrated items from the Aboriginal and Torres Strait Islander QAT and our identified principles of participatory design to create an emerging framework for developing dMH resources with Indigenous young people (Fig. 2). This framework was presented to key research team members (KD, TN, PPJRM) with discussions to refine and ensure all elements of best practice and recommendations were included, appropriately represented, and defined. The scoped literature was then assessed against each element of this framework. The results are presented for each of the four key domains of the model: governance, engagement, partnerships, and research translation. Supplementary file 4 clarifies each aspect of the framework and presents examples from the scoped literature.

### Best practice approaches and recommendations for dMH design with Indigenous young people

#### Governance

Studies undertaken by Indigenous health services or boards [75, 77, 81, 87, 88] generally had suitable Indigenous governance structures, which were involved in determining priorities and study processes. Other studies embedded in existing research programs [58, 60, 61, 65, 66, 72, 73, 85] described the inclusion of Elders, community members, and tribal review boards, which strengthened their Indigenous governance and leadership processes. In some cases, information was scarce regarding early planning consultation, making it difficult to establish if the research specifically responded to a need identified by the community or if consultation and governance processes were appropriately representative of the community included in the research.

Most studies acknowledged youth diversity and considered representativeness in sample selection or interpretation of results. As a result, almost all studies recruited participants from several sites, with eight specifically targeting rural or remote communities [31, 58, 60, 68–70, 77, 78, 81] or national samples [63, 88]. Author reflection and discussion around power considerations and stakeholder and young people’s involvement in decision-making and research planning was infrequent but evident in at least one study [31]. Two studies described shared decision-making processes with study participants and outlined their voting methods to determine the look and feel of design elements [31, 80].

Most studies demonstrated Indigenous leadership or governance, which resulted in improved engagement [31, 61, 65, 70, 77, 81, 89], tailoring of study processes [31, 61, 65, 90], and likely the success of most projects. In several studies, it was difficult to determine leadership and governance structures. Indigenous leadership was evidenced through the inclusion of Indigenous people in various project roles, including as lead investigators [61, 64, 65], co-authors or investigators [31, 66, 79, 90], reference group members [31, 58, 86], governance boards [75, 77, 87], or as advisors, community elders or key informants [73, 86, 90].

#### Engagement

All studies engaged young people throughout an iterative process of design, development, or review, to some degree, evidenced by prolonged engagement (i.e. multiple workshops or focus groups) [31, 58, 60, 77, 81] or multiple phases [65, 66, 68–70, 78–80, 85]. In some cases, it was difficult to establish the degree of youth involvement in the initial design as the formative design and development phases were not reported [60, 62, 65, 85, 91].

Most studies described experiential, playful action-based activities undertaken to generate, design, or evaluate resources. Focus groups and interviews were predominantly used, with some questionnaires included to complement other methods [58, 61, 86]. Focus group and interview processes generally included viewing or using a dMH intervention, followed by group discussion or questioning. Two studies provided a detailed description of workshop activities [31, 58] that included discussion, vignettes, photovoice methods, body mapping activities, review, discussion of dMH tools and features, and co-analysis with participants. Two studies showed multiple websites and dMH programs to further illicit ideas and create a discussion based on user preferences [58, 81].

All studies made adaptions to respect local cultural protocols, leading to increased recruitment [78, 88], quality of data collected [31, 90], and acceptability of study outcomes and processes [61, 65, 75, 81, 86, 88]. Several studies considered Indigenous constructs of wellbeing [61, 65, 70, 78], Indigenous language groups [58, 70], family groups [61], or tribal affiliations [71] when planning study procedures to ensure these aligned with community protocols. Examples included the use of interpreters [58], gender considerations [58, 62], cultural introductions or greetings [61, 65], rapport building exercises such as providing food [65, 73], and adapting data collection tools [60, 61, 65], processes or methodology [70]. One study excluded outcome measures as the measures were considered culturally inappropriate and may increase program dropout [77], others broadened inclusion criteria to allow recruitment of young people with more complex needs [70, 86].

An Indigenous paradigm guided almost all studies. Those who acknowledged the holistic nature of Indigenous wellbeing also demonstrated consideration of local processes. The use of Indigenous methodologies or the involvement of Indigenous people in data analysis and interpretation [58, 61, 64, 65, 85, 90] were identified to strengthen the trustworthiness of the data collected. Two other studies robustly justified the choice of methodology and its application in an Indigenous setting [12, 81] while also relying on local staff to guide study implementation, which led to high acceptability of study processes and engagement from the community. One study reflected in depth regarding the challenges of undertaking their work within a ‘Western framework’ and highlighted how these learnings ‘will allow for a more culturally competent and rigorous approach to conducting future research’ [70]. Most studies described a strengths-based approach to research, evidenced through research aims, activities, and reporting.

#### Partnerships

Partnerships were most often demonstrated through capacity strengthening activities. Several studies included specific statements reflecting the respect shown, knowledge gained, or training provided to young people and other stakeholders to ensure their meaningful engagement [60, 65, 69, 75, 86]. These studies provided high-quality examples of participatory design and were most likely to reflect on their engagement processes consistent with best-practice research with Indigenous young people. There were examples of youth participants upskilling in technology use [62], video production [77, 81], and research roles (peer researcher, mentor, analysis) [58, 81, 86]. Seven studies reported procedures to ensure the participant's safety, including risk management procedures [58, 60, 64, 78, 81]. Several studies reported agreements with local education and support services to support at-risk individuals [58, 60, 70]. Other examples of capacity strengthening included the employment of local staff [31, 73, 77, 81, 90] and specific strategies to engage and train Indigenous academics [63, 91].

Other study processes which demonstrate successful partnerships between participants and researchers were less frequently reported in the academic literature. Only one study highlighted the tensions between stakeholder groups (i.e. end-users, service providers, literature, and researchers) and identified strategies to enhance their processes [75]. Five studies discussed the challenges and implications of funding, budget, and timeframe restrictions on project scope [31, 68, 70, 75, 80], with none reporting on how they managed participant or stakeholder expectations throughout this process.

Rarely did studies detail agreements to protect existing or created Indigenous knowledges with participating individuals, communities, or organisations [53, 70]. Some studies highlighted that these agreements were part of the tribal review board processes [75, 87]. However, as processes differ within the local, national, and international context, it was difficult to establish if control over existing and created intellectual property was included in review board agreements.

#### Knowledge translation

All studies designed, developed, or evaluated dMH tools to improve mental health outcomes for Indigenous young people, demonstrating some intended benefit to the communities involved. Most studies planned to or did translate findings into sustainable changes in policy or practice. In some cases, the ongoing benefit was difficult to establish, as it was unclear if the intervention was implemented beyond the research presented. Of the 11 interventions examined, to our knowledge, five are currently publicly available (SPARX, ibobbly, BRAVE, Stayin' on Track, and AIMhi-Y), with two others implemented across several universities or clinics (e-SIB, YouthCHAT). Identification and follow-up for distress or risky behaviours, delivery of a dMH intervention [58, 60, 62, 64, 66, 70, 72], psychoeducation [71, 85], skill development [58, 62, 77, 81, 86], and a sense of pride or empowerment [77, 81] were other benefits identified for, or by, individual participants.

All studies reported outcomes of their design, development, or evaluation of dMH tools. Most dMH programs described their design and development phase; however, the depth of reporting varied significantly, leading to challenges in identifying the source, progression, and depth of youth involvement throughout. Participant evaluation of study processes was infrequently reported, making it difficult to determine the acceptability of research processes and outcomes from the perspective of Indigenous young people. Six studies evaluated Indigenous young people’s involvement in the research processes, using rating scales [61, 86], an evaluation survey [77], and exit interviews [69, 77, 81, 86]; each reported favourable young persons' perceptions of involvement in the project. Detailed dissemination strategies for individuals and community members were rarely included in the published literature. Those who did discuss dissemination used strategies including community meetings [77, 80, 81], feedback to service providers [63, 70], and social media communication [58].

## Discussion

This review has examined the processes used to involve Indigenous young people in designing and evaluating dMH interventions and examined how methods align with best practice for undertaking Indigenous health research and recommendations for participatory design derived from the literature. In recognition of the similarity between principles in these areas, we present an integrated framework for developing and evaluating dMH resources with Indigenous young people (Fig. 2). Overall, most studies demonstrated governance, engagement, and knowledge translation to a high quality with practice examples outlined (Supplementary file 4). However, demonstrated evidence of quality partnerships were less frequent in the academic literature. This may be due to the concise nature of academic writing and the complexities of negotiating a respectful, reciprocal, and relational co-design process within a cross-cultural context [92]. Overall, reporting was inconsistent and required several sources to discern information, which continues to hamper efforts to advance participatory methodologies [29] and determine the impact of collaborative design and evaluation on dMH uptake and effectiveness [46]. Several areas for potential improvement were identified.

Our findings regarding inconsistent reporting align with other findings examining dMH development and evaluation processes [20, 21, 93]. Detailed reporting of sample demographics, co-design processes, and participants’ subjective experiences of using dMH tools has been recommended to improve understanding of individual user types and engagement styles [21, 47]. Improved knowledge—of what works, for whom, and why—could facilitate the development of tailored tools and targeted implementation efforts to engage particularly hard-to-reach Indigenous young people (i.e. male, English as a second language, justice settings, or severely unwell) [20, 21].

Detailed reporting of research processes, in line with our presented framework of recommendations, would assist in the development of clear methodological approaches [29] and improve the transparency of culturally responsive research practices [35]. Specific reporting gaps identified through this scoping study include: Indigenous governance structures, intellectual property agreements, group cohesiveness, decision-making strategies, short- and long-term benefits, and the acceptability of study processes from young people's perspectives. These factors have the potential to influence co-design outcomes, the feasibility, and the value of developed dMH approaches [94].

This review has several strengths. By examining the literature on processes used to involve Indigenous young people in dMH design and evaluation and assessing these against best practice approaches in Indigenous research and emerging principles of participatory design, we have highlighted strengths and weaknesses in current practice and reporting. Drawing on these findings and existing Indigenous research and participatory design guidelines and recommendations, we present an emergent framework to guide future research (Fig. 2). Nevertheless, several limitations exist. As noted by others, this emerging field often lacks consistency in terms used for reporting dMH interventions [33], so it is possible references were missed. Publication bias may have also impacted the studies identified, as inconclusive or negative findings may not have been published. The assessment of alignment with best practice guidelines was conducted using only a desktop review with limited or no author communication in some instances, relying on reporting rather than study conduct. We acknowledge that most studies are likely to have implemented additional activities in line with best practice that were not reported. Furthermore, we assessed studies against the Aboriginal and Torres Strait Islander QAT [45], a guideline developed in Australia, potentially biasing assessment of studies from other countries. However, this is the only available quality appraisal tool for Indigenous research internationally [45] and our assessment of the tool’s alignment with other international guidelines demonstrated that it served as an adequate tool within this study. We support the developers’ plans for the tool to be assessed and revised to suit a global context [45].

This review has identified several potential areas for improving the current evidence base. Future research should be reported in a standardised format to ensure transparency, quality, and advancement of dMH development and evaluation methodologies. Reporting guidelines such as the Aboriginal and Torres Strait Islander QAT [53] and the Consolidated criteria for reporting qualitative research (COREQ) [95] provide valuable tools. Reporting against the participatory design principles, derived from the literature, described within this study and the emergent framework integrating these principles with elements of the QAT will also enhance learning and aid future research attempts to engage Indigenous young people and other minority groups in such projects.

Several benefits of including Indigenous young people in the design or evaluation of the dMH tools were noted, including improved engagement [75, 87, 88], acceptability of developed resources [77, 81], and ongoing program support [31, 58, 69, 81]. Engaging young people in project roles through employment, reference groups, or participatory action research roles provided opportunities for skill development and education [86]. These strategies assist in privileging Indigenous young people's voices and provide opportunities for addressing the health and social disadvantages they face through empowerment [96]. This aligns with best practice guidelines in that the benefits of involvement should extend beyond the individual involved in the research to family, community, and the wider population [53]. Furthermore, Indigenous young people’s holistic worldviews of well-being offer unique opportunities for practical implementation. A greater understanding of the potential role of family and community in supporting the development and use of dMH tools is required to assist future implementation efforts [64, 70]. Lastly, due to long timeframes in developing and testing quality dMH interventions, projects need to be community-led and embedded in existing programs, with reputable and longstanding collaborations and funding. It is also imperative that dMH tools continue to evolve to keep up with rapid technological advancements and changes in youth culture and attitudes toward dMH tools. There is potential for international collaborations for dMH design (e.g. SPARX and iSPARX) to overcome some of these challenges, by recognising and building on similarities across some Indigenous cultures worldwide and adapting and re-modelling programs to suit local need. Furthermore, methodologies for evaluating and implementing dMH tools must also adapt to accommodate rapid evaluation and translation into practice to avoid such tools becoming outdated or obsolete by the time they are validated [97].

## Conclusions

This review has identified gaps in the reporting of dMH intervention development and evaluation studies for Indigenous young people. Until we have best practice guidelines for participatory design and consistency of reporting, the strength of evidence regarding the effects of participatory design on uptake and outcomes in dMH will remain limited. This study has integrated best practice recommendations for Indigenous research and recommendations for participatory design to develop an emergent framework for the engagement of Indigenous young people in dMH development or evaluation. Common strengths of the reviewed studies included the adaption of study processes to engage Indigenous young people, involvement of Indigenous people in research processes, and capacity strengthening. Common gaps included the lack of transparent reporting regarding sample representativeness, intellectual property agreements and limited progression into implementation studies. Consistent and detailed reporting is needed within this developing field to ensure that the opportunities presented by dMH, especially for hard-to-reach populations, are realised.

## Supplementary Information

**Additional file 1.** 

**Additional file 2.** 

**Additional file 3.** 

**Additional file 4.** An emerging framework for the development or evaluation of dMH resources with Indigenous young people: Description and examples from the scoped literature.  

## Data Availability

The materials supporting the article are included as Supplementary files 1, 2, 3and 4.

## Abbreviations

dMH: Digital Mental Health
PD: Participatory design
