# Supplementary material for: An emerging framework for digital mental health design with Indigenous young people: a scoping review of the involvement of Indigenous young people in the design and evaluation of digital mental health interventions
Source: Syst Rev. 2023 Jul 1;12:108. doi: 10.1186/s13643-023-02262-w (PMC10314399; doi:10.1186/s13643-023-02262-w)
Supplement: Supplementary file 2 — Additional file 2. [file 13643_2023_2262_MOESM2_ESM.docx]

# Supplementary file two:

## Search Strings per database

### EBSCOhost databases

Search Date: 18/9/2020, 22/02/2022 & 3/3/2023

| Databases | EBSCOhost databases (Academic Search Premiere, Computer and Applied Science complete, CINAHL Plus with Full text, MEDLINE with full text, APA PsychArticles, Psychology and Behavioural Sciences collection, APA PsychInfo) |
| --- | --- |
| Search String | (“aboriginal and torres strait islander” or aboriginal or “first people” or “first nation people” or “first nation*” or indigenous or “Indian American” or metis or native or inuit or maori OR pacific* OR hawai* or Alaskan OR “Oceanic ancestry group”) AND (youth or adolescen* or teenage* or “young people” or “young adult” or child*) AND (“e-Mental Health” OR e-Health OR “electronic health” OR “digital mental health” OR telemedic* OR telehealth OR “telemental health” OR mhealth OR “mobile health” OR mtherapy OR “online therapy” OR “online intervention” OR e-therapy OR “internet intervention” OR “computer-assisted therapy” OR “online self-help” OR iCBT OR “website intervention” OR “web-based treatment” OR “serious game*” OR SMS OR “text messaging” OR “text message” OR “distance based intervention” OR “internet-based intervention” or “teleintervention” OR “MH-App” OR “mental health app” OR “computerised therapy” OR “computerized therapy”) AND (“mental health” or psych* or wellbeing OR well-being OR distress OR depression OR anxi* OR phobia OR mental OR mood OR suic*) |
| Limits | 1990-Current |
| No. Identified | 510 |
| No. exported | 510 |

### PubMed

Search Date: 18/9/2020, 22/02/2022 & 3/3/2023

| Search String | ("aboriginal and torres strait islander"[All Fields] OR ("aboriginal"[All Fields] OR "aboriginality"[All Fields] OR "aboriginals"[All Fields] OR "aborigine"[All Fields] OR "aborigines"[All Fields]) OR "first people"[All Fields] OR "first nation people"[All Fields] OR "first nation*"[All Fields] OR ("indigene"[All Fields] OR "indigeneity"[All Fields] OR "indigeneous"[All Fields] OR "indigenes"[All Fields] OR "indigenization"[All Fields] OR "indigenous"[All Fields]) OR "Indian American"[All Fields] OR "metis"[All Fields] OR ("native"[All Fields] OR "native s"[All Fields] OR "natively"[All Fields] OR "nativeness"[All Fields] OR "nativity"[All Fields] OR "population groups"[MeSH Terms] OR ("population"[All Fields] AND "groups"[All Fields]) OR "population groups"[All Fields] OR "natives"[All Fields]) OR ("inuits"[MeSH Terms] OR "inuits"[All Fields] OR "inuit"[All Fields]) OR ("maori"[All Fields] OR "maoris"[All Fields]) OR "pacific*"[All Fields] OR "hawai*"[All Fields] OR ("alaskan"[All Fields] OR "alaskans"[All Fields]) OR "Oceanic ancestry group"[All Fields]) AND ("adolescent"[MeSH Terms] OR "adolescent"[All Fields] OR "youth"[All Fields] OR "youths"[All Fields] OR "youth s"[All Fields] OR "adolescen*"[All Fields] OR "teenage*"[All Fields] OR "young people"[All Fields] OR "young adult"[All Fields] OR "child*"[All Fields]) AND ("e-Mental Health"[All Fields] OR "e-Health"[All Fields] OR "electronic health"[All Fields] OR "digital mental health"[All Fields] OR "telemedic*"[All Fields] OR ("telehealth s"[All Fields] OR "telemedicine"[MeSH Terms] OR "telemedicine"[All Fields] OR "telehealth"[All Fields]) OR "telemental health"[All Fields] OR ("telemedicine"[MeSH Terms] OR "telemedicine"[All Fields] OR "mhealth"[All Fields]) OR "mobile health"[All Fields] OR "mtherapy"[All Fields] OR "online therapy"[All Fields] OR "online intervention"[All Fields] OR ("distance counseling"[MeSH Terms] OR ("distance"[All Fields] AND "counseling"[All Fields]) OR "distance counseling"[All Fields] OR "e therapy"[All Fields]) OR "internet intervention"[All Fields] OR "computer-assisted therapy"[All Fields] OR "online self-help"[All Fields] OR "iCBT"[All Fields] OR "website intervention"[All Fields] OR "web-based treatment"[All Fields] OR "serious game*"[All Fields] OR ("smart mater struct"[Journal] OR "sms"[All Fields]) OR "text messaging"[All Fields] OR "text message"[All Fields] OR (("distance"[All Fields] OR "distances"[All Fields]) AND ("based"[All Fields] OR "basing"[All Fields]) AND ("intervention s"[All Fields] OR "interventions"[All Fields] OR "interventive"[All Fields] OR "methods"[MeSH Terms] OR "methods"[All Fields] OR "intervention"[All Fields] OR "interventional"[All Fields])) OR "internet-based intervention"[All Fields] OR "teleintervention"[All Fields] OR "mental health app"[All Fields] OR "computerised therapy"[All Fields] OR "computerized therapy"[All Fields]) AND ("mental health"[All Fields] OR "psych*"[All Fields] OR "wellbeing"[All Fields] OR ("health"[MeSH Terms] OR "health"[All Fields] OR "well"[All Fields] OR "well being"[All Fields]) OR ("distress"[All Fields] OR "distressed"[All Fields] OR "distresses"[All Fields] OR "distressful"[All Fields] OR "distressing"[All Fields]) OR ("depressed"[All Fields] OR "depression"[MeSH Terms] OR "depression"[All Fields] OR "depressions"[All Fields] OR "depression s"[All Fields] OR "depressive disorder"[MeSH Terms] OR ("depressive"[All Fields] AND "disorder"[All Fields]) OR "depressive disorder"[All Fields] OR "depressivity"[All Fields] OR "depressive"[All Fields] OR "depressively"[All Fields] OR "depressiveness"[All Fields] OR "depressives"[All Fields]) OR "anxi*"[All Fields] OR ("phobic disorders"[MeSH Terms] OR ("phobic"[All Fields] AND "disorders"[All Fields]) OR "phobic disorders"[All Fields] OR "phobia"[All Fields] OR "phobias"[All Fields]) OR ("mental"[All Fields] OR "mentalities"[All Fields] OR "mentality"[All Fields] OR "mentalization"[MeSH Terms] OR "mentalization"[All Fields] OR "mentalizing"[All Fields] OR "mentalize"[All Fields] OR "mentalized"[All Fields] OR "mentally"[All Fields]) OR ("affect"[MeSH Terms] OR "affect"[All Fields] OR "mood"[All Fields]) OR "suic*"[All Fields]) |
| --- | --- |
| Limits | 1990-Current;  Filters: Humans, English, Child: 6-12 years, Adolescent: 13-18 years, Adult: 19+ years, Young Adult: 19-24 years, Adult: 19-44 years |
| No. Identified | 2182 |
| No. exported | 2182 |

### Scopus

Search Date: 18/9/2020, 22/02/2022 & 3/3/2023

| Search String | *( TITLE-ABS-KEY ( (*"aboriginal and torres strait islander"*OR*aboriginal*OR*"first people"*OR*"first nation people"*OR*"first nation*"*OR*indigenous*OR*"Indian American"*OR*metis*OR*native*OR*inuit*OR*maori*OR*pacific**OR*hawai**OR*alaskan*OR*"Oceanic ancestry group"*) )  AND  TITLE-ABS-KEY ( (*youth*OR*adolescen**OR*teenage**OR*"young people"*OR*"young adult"*OR*child**) )  AND  TITLE-ABS-KEY ( (*"mental health"*OR*psych**OR*wellbeing*OR*well-being*OR*distress*OR*depression*OR*anxi**OR*phobia*OR*mental*OR*mood*OR*suic**) )  AND  TITLE-ABS-KEY ( (*"e-Mental Health"*OR*e-health*OR*"electronic health"*OR*"digital mental health"*OR*telemedic**OR*telehealth*OR*"telemental health"*OR*mhealth*OR*"mobile health"*OR*mtherapy*OR*"online therapy"*) )  OR  TITLE-ABS-KEY (*"online intervention"*OR*e-therapy*OR*"internet intervention"*OR*"computer-assisted therapy"*OR*"online self-help"*OR*icbt*OR*"website intervention"*OR*"web-based treatment"*OR*"serious game*"*OR*sms*OR*"text messaging"*)  OR  TITLE-ABS-KEY (*"text message"*OR*"distance based intervention"*OR*"internet-based intervention"*OR*"teleintervention"*OR*"MH-App"*OR*"mental health app"*OR*"computerised therapy"*OR*"computerized therapy"*) )* |
| --- | --- |
| Limits | 1990-Current; English, Country: USA, NZ, Australia, Canada |
| No. Identified | 218 |
| No. exported | 218 |

## Grey Literature Searches – delayed till after first 10% kappa rating for abstract screen and refine inclusion criteria

### Informit

Search Date: 18/9/2020 – 12/11/2020, 22/02/2022, 3/3/2023

| Search String | ((&#147;aboriginal and torres strait islander&#148; or aboriginal or &#147;first people&#148; or &#147;first nation people&#148; or &#147;first nation*&#148; or indigenous or &#147;Indian American&#148; or metis or native or inuit or maori OR pacific* OR hawai* or Alaskan OR &#147;Oceanic ancestry group&#148;)) AND ((youth or adolescen* or teenage* or &#147;young people&#148; or &#147;young adult&#148; or child*)) AND ((&#147;e-Mental Health&#148; OR e-Health OR &#147;electronic health&#148; OR &#147;digital mental health&#148; OR telemedic* OR telehealth OR &#147;telemental health&#148; OR mhealth OR &#147;mobile health&#148; OR mtherapy OR &#147;online therapy&#148; OR &#147;online intervention&#148; OR e-therapy OR &#147;internet intervention&#148; OR &#147;computer-assisted therapy&#148; OR &#147;online self-help&#148; OR iCBT OR &#147;website intervention&#148; OR &#147;web-based treatment&#148; OR &#147;serious game*&#148; OR SMS OR &#147;text messaging&#148; OR &#147;text message&#148; OR &#147;distance based intervention&#148; OR &#147;internet-based intervention&#148; or &#147;teleintervention&#148; OR &#147;MH-App&#148; OR &#147;mental health app&#148; OR &#147;computerised therapy&#148; OR &#147;computerized therapy&#148;)) AND ((&#147;mental health&#148; or psych* or wellbeing OR well-being OR distress OR depression OR anxi* OR phobia OR mental OR mood OR suic*)) |
| --- | --- |
| Limits | 1990-2020; |
| No. Identified | 17406 (limited to first 200) |
| No. exported | 0 |

### Google

Search Date: 12/11/2020, 22/02/2022 & 3/3/2023

| Search String | (“aboriginal and torres strait islander” OR aboriginal OR “first people” OR “first nation people” OR “first nation*” OR indigenous OR “Indian American” OR metis OR native OR inuit OR maori OR pacific* OR hawai* or Alaskan OR “Oceanic ancestry group”) AND (youth or adolescen* or teenage* or “young people” or “young adult” or child*) AND (“e-Mental Health” OR e-Health OR “electronic health” OR “digital mental health” OR telemedic* OR telehealth OR “telemental health” OR mhealth OR “mobile health” OR mtherapy OR “online therapy” OR “online intervention” OR e-therapy OR “internet intervention” OR “computer-assisted therapy” OR “online self-help” OR iCBT OR “website intervention” OR “web-based treatment” OR “serious game*” OR SMS OR “text messaging” OR “text message” OR “distance based intervention” OR “internet-based intervention” or “teleintervention” OR “MH-App” OR “mental health app” OR “computerised therapy” OR “computerized therapy”) AND (“mental health” or psych* or wellbeing OR well-being OR distress OR depression OR anxi* OR phobia OR mental OR mood OR suic*) |
| --- | --- |
| Limits | nil |
| No. Identified | 1,370,000+ results (limited to first 200) |
| No. exported | 0 |
