# Supplementary material for: An emerging framework for digital mental health design with Indigenous young people: a scoping review of the involvement of Indigenous young people in the design and evaluation of digital mental health interventions
Source: Syst Rev. 2023 Jul 1;12:108. doi: 10.1186/s13643-023-02262-w (PMC10314399; doi:10.1186/s13643-023-02262-w)
Supplement: Supplementary file 3 — Additional file 3. [file 13643_2023_2262_MOESM3_ESM.docx]

# Supplementary File Three: Data Extraction Variables

| **Variable** | **Details and examples** |
| --- | --- |
| Study details | Authors, date, title, journal, volume, issue, pages, country of origin, aim/objective, context |
| Description of the digital mental health resource | Purpose, technology type, target population, service type, therapeutic basis, mode of delivery |
| **Co-design or evaluation processes** | |
| Stage of development or evaluation | Predesign (formative), early design, post prototype, feasibility, efficacy or effectiveness trial, implementation |
| Study design | Participatory design, co-design, pilot study, randomised controlled trial |
| Participant demographics | Number, age, gender, ethnicity, languages spoken, English proficiency, diagnosis, role (i.e., student, patient, carer, health professional type) |
| Data collection | Number, duration, and mode (i.e., face to face, internet) of participant contacts, sample size, sites of data collection (e.g., School, community service), methods used (e.g., focus groups, workshops, interviews, age-appropriate experiential, fun or playful activities), support personnel included (e.g., interpreters, support staff) |
| **Benefits to the community or individual** | |
| Justification for project and source | Literature, community consultation, previous formative study, or pilot |
| Building capability (individual and community) | Employment of Indigenous staff, community engagement/information sessions, professional development opportunities for staff or participants |
| The benefit of the research | Results, outcomes and future intentions, consideration or evidence of the benefits to participants and the broader community |
| Dissemination practices | To whom, when, platforms used |
| **Consultation and participation of Indigenous people** | |
| Training and resources provided to Indigenous research participants to promote participation | Training in suicide prevention to aid design processes, videography workshops, peer researcher training, lending devices, upskilling participants throughout co-design activities |
| Safety considerations | Detailed risk management plans |
| Participant feedback on design or evaluation processes | Exit interview data or rating scales of acceptability |
| Frank discussion of design or evaluation processes (strengths and limitations) | Transparent decision-making processes, process reporting and reflection, managing diversity of the sample, tensions between participant groups and therapy and pedagogy experts |
| **Respect and recognition of Indigenous knowledges and practices** | |
| Research processes that consider the physical, social, economic, and cultural environment of participants | Community consent processes, interpreters, following local cultural protocols (i.e., greetings & rituals), permission for research/involvement of Traditional Owners/Tribal leaders, involvement of local delegates |
| Reporting and analysis which considers the physical, social, economic, and cultural environment of the participants | Consideration of social determinants of health, strengths-based reporting, presentation, and consideration of differing worldviews in reporting and analysis |
| The rationale of methods/methodologies used | Literature, previous research, alignment with Indigenous worldviews |
| Consent processes reported | Individual, parent, or collective consent, online or face to face, parties involved in the consenting process (interpreter, support person) |
| **Indigenous self-determination and governance** | |
| Partnerships with Indigenous corporations or communities | Memorandum of Understandings, negotiation processes, agreements reached & reviewed, approvals or agreements with location-specific health or governance boards, advisory boards, funding controls |
| Ethics board clearances | Indigenous health research ethics committees |
| Data ownership | Agreements of existing and created data ownership/intellectual property reported |
