# Supplementary material for: An emerging framework for digital mental health design with Indigenous young people: a scoping review of the involvement of Indigenous young people in the design and evaluation of digital mental health interventions
Source: Syst Rev. 2023 Jul 1;12:108. doi: 10.1186/s13643-023-02262-w (PMC10314399; doi:10.1186/s13643-023-02262-w)
Supplement: Supplementary file 4 — Additional file 4. An emerging framework for the development or evaluation of dMH resources with Indigenous young people: Description and examples from the scoped literature. [file 13643_2023_2262_MOESM4_ESM.docx]

# Supplementary file four:

## An emerging framework for the development or evaluation of dMH resources with Indigenous young people: Description and examples from the scoped literature

| Principle | Description | Examples |
| --- | --- | --- |
| **Governance** |  |  |
| Address a need identified by community **(QAT Q1)** | Ensure the project is supported by the community. Consult community leaders, elders, family, service providers and young people. | e.g. ‘Project Life was developed with Maniilaq Association, a tribal health and social services organization serving NW Alaska. The project was funded by a grant to Maniilaq… In the evaluation, we worked with our tribal partners to figure out what questions were needed… So, the project was based in a tribal health organization, the staff worked for that organization, and the funding went to that organization…’ (Project life; Wexler, L, personal communication, Nov 2021)  e.g. ‘Inuit wisdom and the scientific literature emphasise the need for any intervention to integrate evidence-based approaches with Inuit-specific cultural practices’ (iSPARX; Litwin, 2023) (1) |
| Ensure consultation and decision making is appropriately inclusive **(QAT Q2; PD Q5 & 6)** | Identify relevant stakeholders and ensure representation is appropriately inclusive.  Ensure decision making processes are described. Power relations are considered, and processes reflected on. Suggestions for improvement maybe presented. | e.g. ‘The Northwest Portland Area Indian Health Board (NPAIHB) is a tribal organization that represents 43 federally recognized tribes in Washington, Oregon, and Idaho (NW)… [the] governing board meets quarterly and is composed of one delegate from each member tribe, selected by the individual tribal governments… This formative research was a collaboration between the NPAIHB’s THRIVE and We R Native projects, [and] ….’ ‘[we] pilot test[ed] the intervention with 16 AI/AN young men with a history of alcohol use and violence, and with 8 topical experts in alcohol prevention, alcohol treatment, violence prevention, health communication, and adolescent health.’ (BRAVE; Rushing, 2021)(2)  e.g. ‘Each co-design workshop was iteratively modified in response to previous findings, limiting the use of voting or consensus methods [38,37]. Therefore, informed by previous findings, literature, and safety standards, researchers assumed a decision-making role, which shifted power differentials and impacted the extent of young people’s influence in the co-design process. The sustained and regular inclusion of 2 youth and 2 senior Indigenous researchers and an experienced research team optimized youth, clinician, and Aboriginal and Torres Strait Islander influence in decision-making. The inclusion of the IYRG throughout development improved the decision-making capacity of young people in some domains. However, the information and decisions presented to the group were prioritized by the research team to keep within time frame constraints. Systematically taking findings and decisions from one group to the other and back again (e.g., through decision logs) might have improved the transparency of our decision-making.’ (AIMhi-Y; Povey, 2022)(3) |
| Demonstrate Indigenous research leadership **(QAT Q3 & 4)** | Include Indigenous CI’s and Indigenous governance structures (reference groups, tribal groups). Respect cultural processes specific to region, area, country. | e.g. ‘We had Te Roopu Māori on the steering group for the study, we had a senior Māori researcher on the investigator team, and we had Māori emerging researchers undertaking Māori -focused development research, consistent with a Kaupapa Māori approach.’ (e-SIB; Kypri, K, Personal communication, Nov 2021)  e.g. ‘As well as the Māori (young people) … the research team had a kaumatua (elder) with content knowledge and cultural knowledge and connections…  he took this to iwi (tribal) leadership groups and shared his own insights. [Additionally] the computer games company… is a Māori led company… with Māori [director] and she led all the programming…’, (SPARX; Fleming, T, personal communication, Nov 2022)  e.g. ‘One male and two female Senior Indigenous Co-Researchers played an integral role in study design, consent, data collection, analysis and dissemination ensuring the study was informed by Aboriginal and Torres Strait Islander perspectives throughout. An Expert Reference Group (ERG) provided guidance and advice on study procedures… ERG membership (25% Male, 42% Aboriginal), represented most states in Australia.’ (AIMhi-Y; Povey 2020)(4) |
| **Engagement** | | |
| Engage young people in an iterative process of design, development and review **(PD Q1)** | Young people should be engaged in an iterative process of design, development, and review. A flexible and timely process can aid ongoing engagement. | e.g. ‘The intervention was developed iteratively over a 10-year period involving consultation with Ma¯ori and non-Ma¯ori university students, Ma¯ori student support services and with the aid of Ma¯ori co-investigators and research staff. (e-SIB; Kypri, K, personal communication, Nov 2022)  e.g. A series of five or six co-design workshops (29 in total) was conducted five times, within two schools and a drug rehabilitation service… A closed Social Media Group complemented each face to face co-design workshop group… An iterative process of data analysis, feedback and member checking with participants occurred throughout the series of co-design workshops. (AIMhi-Y; Povey 2020)(4) |
| Acknowledge youth diversity **(PD Q2)** | Acknowledge differing preferences between seemingly similar users. Design interventions to effectively integrate suggestions while balancing the desire to develop something that aims to suit everyone. Highlight limitations. | e.g. The BRAVE formative research had several strengths… [including] the diversity of AI/AN young adults involved… BRAVE’s focus on culturally relevant images, language, and the use of peers as role models demonstrated respect for diversity, values, and translation of health communication messaging that AI/AN youth could identify with… Participants represented 10 communities and did not represent the geographic and tribal diversity of all AI/AN young adults in the US. (BRAVE; Rushing, 2021)(2)  e.g.…‘we also made effort to include those who were disengaged from school resulting in a wide range of participants from varying age groups, socioeconomic circumstances, geographical locations and language backgrounds. In doing so, we hope that the developed app will be relevant and appealing to a wide variety of young people.’ (AIMhiY; Povey 2020)(4) |
| Plan fun and experiential activities that respect age and cultural protocols **(PD Q3; QAT Q5)** | Design and evaluation processes need to be suited to the age and cultural background of the young people involved. Rapport building, knowledge generation and data collection activities should reflect local protocols. Include peers, Elders, family and other support people where appropriate to recognise the holistic nature of Indigenous wellbeing. | e.g. ‘The first author, MS, is Māori and ensured that all study processes took into account Māori processes and protocols… MS was welcomed with a powhiri (formal welcome ceremony) when conducting the focus groups. He responded with a mihi (formal speech), after which waiata (traditional Māori song/s, which had been specially chosen to fit the context) were performed and kai (food) was then shared… The taitamariki group was conducted with young people from a kapa haka group (Māori youth traditional performing arts group)… [and] held at the kapa haka group’s school marae (sacred meeting place)… interviews with wh nau (families) were held in wh nau homes. Wh nau involvement in all aspects of life, including mental well-being, is important for Māori, and is considered one of the four cornerstones of Māori holistic well-being [30]. The inclusion of feedback from wh nau was thus seen as particularly important. Māori protocols (tikanga) were followed when conducting these interviews [29]..’ (SPARX; Shepherd 2015)(5)  e.g. ‘The first author… and a male/female Indigenous Research Officer, who shared the same language background as the participants, facilitated the co-design workshops to ensure cultural safety and enable engagement using participants' first language.’ (AIMhi-Y; Povey 2022)(4)  e.g. ‘The interview questions were reviewed and approved by Indigenous mental health professionals… Interviews were not recorded, as recording Indigenous youth speaking about their own suicidality with an unfamiliar non-Indigenous researcher was regarded as potentially uncomfortable for participants.’ (Ibobbly; Tighe 2020)(6) |
| Ensure methodological rigour and fit **(QAT 9 & 10)** | Be aware of the various contexts (i.e. historical, political, local) in which the research will occur. Use a strengths-based Indigenous research paradigm to guide study design. Involve Indigenous people in study design, data collection and analysis. Carefully consider your methodology and provide adequate description of its fit within this context. | e.g. ‘In addition, a third author (TM), an Indigenous community member, ensured all authors were representing the cultural aspects of the themes as accurately as possible’ (ibobbly; Tighe, 2020)(6)  e.g. ‘All of the NPAIHB’s research and public health programs are guided by principles of community-based participatory research (CBPR)… CBPR was appropriate for this study because it focuses on AI/AN young adults as experts, acknowledges cultural influences, and identifies elements of substance misuse, violence, and aggression that are relevant to Native young adults and communities’ ‘Native teens and young adults are indeed brave. They come from a long line of ancestors who fought for freedom, defended their culture and homelands, and relied upon kinship systems and community support to live a healthy life. Formative research from BRAVE builds on this history and connects AI/AN teens and young adults to people, stories, resources, and teachings that demonstrate what it means to be strong and resilient.’ (BRAVE; Rushing, 2021)(2)  e.g. ‘I reviewed the transcripts from focus groups comprising mostly Maori and/or Pacific young people with Maori and Pacific clinicians/researchers to help ensure issues of cultural relevance were not overlooked.’ (SPARX; Fleming, 2012)(7)  e.g. ‘It is important to also note that research that has been conducted in the past has often been detrimental to Maori communities [22]. The history of research within New Zealand has predominantly reflected a distinct patriarchal process in which the Maori worldview has often been marginalized [23]. In this research, we used a Kaupapa Maori approach to ensure that the method of engagement was inclusive of Maori input and respected Maori protocols.’ (SPARX; Shepherd 2015)(5)  e.g. ‘A community-based participatory approach, guided by *Inuit Qaujimajatuqangit*, will be used. As per OCAP guidelines, a collaboration between participants and researchers ensures that community stakeholders have ownership of a project designed to meet their needs, and that research methods are collaborative, culturally embedded, appropriate, and ethical. (iSPARX; personal communication, Bohr & Hankey, 2023) |
| **Partnership** | | |
| Create an environment of mutual learning to ensure capacity strengthening **(QAT 13 & 14; PD Q4)** | Include experts from many backgrounds (youth, researchers, staff, IT experts). Provide opportunities for everyone to learn from each other. Respect, upskill, empower and keep all participants safe throughout the process. | e.g. ‘We recognize that experts exist in all places. Formative research requires teams to seek out this expertise and create a collective knowledge base that honours the unique knowledge, skills, and histories of the target population. In this study, it was AI/AN teens and young adults and tribal communities. The equitable involvement of communities and stakeholders in the formative research process may increase the likelihood that an intervention will be successful. BRAVE formative research viewed feedback from AI/AN teens and young adults as equal to feedback from content experts.’ (BRAVE; Rushing, 2021)(2)  e.g. ‘Twenty young Aboriginal fathers were invited to participate in the project as co-investigators and collaborators, and were paid fees dependent on their level of involvement in the project… Additionally, the project had provided the young fathers with opportunities to network with other fathers and to discuss their common issues; the project was also instrumental in their development as mentors for other young men in the community… Key to the success of the project was the close research partnership that was developed with the Aboriginal communities, and the involvement of the two Aboriginal mentors who supported the young Aboriginal fathers. These young men had an integral role in developing the website content and ensuring that it would be culturally relevant to others experiencing the challenges of fatherhood.’, (Staying on Track; Fletcher 2017)(8)  e.g. ‘The research team… included three First Nations youth researchers studying education and training certificates in community health research, community services and/or Business, a senior cultural advisor and Larakia nation traditional owner as well as non-Indigenous project manager and three non-indigenous senior researchers. The First Nations youth researchers were trained in study procedures and supported by the project manager with clinical mental health experience. Although involved throughout all stages of the study, the youth researchers played a key role in refining study procedures and leading engagement, consent, and data collection with young people’ (AIMhi-Y; Dingwall, 2023) (9) |
| Develop strategies to address predictable tensions **(PD Q6)** | Authors acknowledge tensions exist between end users, and stakeholders. If identified, strategies to manage are outlined. | e.g. ‘Another key challenge was the differing youth preferences highlighted among individuals across and within co-design workshop groups… Although seeking diverse opinions undoubtedly strengthened our approach, a detailed plan of how to integrate the information across stakeholder groups would have aided our process. Identifying stakeholder roles, opportunities for input, responsibilities, influence, and decision-making strategies across app characteristic domains may have allowed more decision-making opportunities to be presented to young people.’ (AIMhi-Y; Povey 2022)(3) |
| Manage expectations **(PD Q7)** | Acknowledge program limitations and challenges. Detail strategies used to manage expectations. | e.g. ‘Formative research requires commitments that withstand the test of time – changes to the target audience, health focus, team members, and funding agency occurred over the five year study. Formative research must be funded, but there are limited resources available in AI/AN communities to support this iterative process. BRAVE pulled funding from multiple sources; this required changing the intervention’s focus, target population, and intervention strategy over time…’ (BRAVE; Rushing 2021)(2) |
| Negotiate intellectual property and control **(QAT 6, 7 & 8)** | Include a clear description of intellectual property agreement and outline participant and community control over data collected. | e.g. ‘All research carried out by the Northwest Portland Area Indian Health Board is reviewed and approved by a Tribal IRB, which actively guides our data collection/protection procedures: [https://nativedata.npaihb.org/](https://aus01.safelinks.protection.outlook.com/?url=https%3A%2F%2Fnativedata.npaihb.org%2F&data=04%7C01%7CJosie.Povey%40menzies.edu.au%7Cf9dacef03d9943de20c708d9a3397849%7C9f2487678e1a42f3836fc092ab95ff70%7C0%7C0%7C637720290723249203%7CUnknown%7CTWFpbGZsb3d8eyJWIjoiMC4wLjAwMDAiLCJQIjoiV2luMzIiLCJBTiI6Ik1haWwiLCJXVCI6Mn0%3D%7C3000&sdata=gM%2BTMJ8TgXzSAmebSBq4KHvzCpYrnE0xZzfZOpPx3u8%3D&reserved=0)’’ (BRAVE; Rushing, S, personal communication, Nov 2022)  e.g. ‘Intellectual property created was not mine/university's. The young people who created digital stories decided whether they wanted to share their stories with the public in community screenings and on the website. Their stories belong to each one of them. Not all stories were shared.’ (Project Life; Wexler, L, personal communication Nov 2022)  e.g. ‘Formal research agreements were established between the York research team and the Nunavut Research Institute. Separate research agreements will be drafted based on each community’s Elders’ council’s recommendations.’ (iSPARX; Bohr & Hankey, J, personal communication, Feb 2023) |
| **Knowledge translation** | | |
| Demonstrate beneficial outcomes for individuals and community **(QAT 12)** | Clearly outline the benefit of the research to the communities involved. The generation of new resources, empowerment and capacity strengthening are some examples. | e.g. ‘The app is a new and novel way of delivering mental health interventions in communities and departs from the traditional modes of face to face delivery. Whilst not a complete solution to suicide prevention within Indigenous communities, the overwhelming acceptability of the app has the potential to change delivery of mental health care, particularly for those remote communities who may not have consistent access to mental health professionals on the ground. Indeed, one of the future uses of the app could be as an adjunct to treatment for those individuals who may only have the opportunity to see mental health professionals on an ad hoc basis. The RCT will help to further detail what impact the app has on mental health in the wider community.’ (bobbly; Black Dog Institute, 2015)(10) |
| Evaluate and disseminate process and outcomes **(PD Q8 & 9)** | Evaluate and report on outcomes and processes of the research. Seek and report young people’s views on involvement. Disseminate research findings widely and detail during reporting. | e.g. ‘As we demonstrated, the digital storytelling project offered young people a way to gain a sense of personal mastery and achievement, highlight positive aspects of themselves, their lives and their reasons for living, as well as offering them a venue for strengthening connections with people that matter. Thus, digital storytelling presents a promising approach to health promotion and primary prevention that can be used to bolster cultural and identity-based protective factors needed for ushering marginalized young people into healthy adulthood.’ (Project Life; Wexler 2013)(11)  e.g. ‘Wh nau are important for the well-being of young people [39]. This study is the first to take these opinions into account in the development of a cCBT program… Traditional M ori (and other indigenous) families will want to be a part of how their young person engages with a cCBT self-help resource… family resources need to be developed alongside a cCBT program so that information is provided to the family about what program their young person is using. This collectivist approach to cCBT and its delivery contrasts considerably with the often individualistic focus of cCBT delivery to date.’ (SPARX; Shepherd, 2015)(5)  e.g. ‘With these barriers and limitations in mind, we are also aware that this pilot study is rooted in a positivistic Western framework and was designed, conducted, and facilitated primarily by Qallunaat (non-Inuit). However, due to limited funding this commissioned study had to be conducted remotely and included directives from the Government of Nunavut to involve local mental health workers, most of whom are not Inuk themselves, to administer the SPARX trial. This exploratory pilot served as a feasibility study fostering enthusiasm and cohesion among participating communities and scaffolding more culturally appropriate research. In a companion study [46] participating youth and community facilitators built much of the knowledge necessary through exit focus groups to facilitate such an endeavour. Indeed, efforts are now well underway to follow up with a more community directed and culturally meaningful study focused on articulating Inuit knowledge and research epistemologies through holistic, relational perspectives rooted in Piliriqatigiinniq, the Inuit concept for working collaboratively for the common good [20].’ (iSPARX; Bohr, 2023) (12) |
| Plan for sustainable change in policy or programs **(QAT 11)** | Clearly identify the planned strategy or next step toward implementation. Articulate outcomes. | e.g. ‘SPARX… was tested with 187 young people from around Aotearoa New Zealand. Our research showed that it helped those that were feeling down, depressed or angry to feel better over time by teaching them self-help skills. SPARX has been freely available within Aotearoa New Zealand since 2015.’ (SPARX website, 2022)  e.g. ‘Since the trial end-date, clinic staff continue to use YouthCHAT and have recommended it to other youth centres in the region. Planning is now underway for further implementation and upscaling into other youth clinics, tailoring the generic framework to specific contexts… the researchers receive regular enquiries for the use and adaptation of the eCHAT programme in a variety of clinical and community settings, both within New Zealand and internationally... Copyright for the programme sits with the researchers at the University of Auckland. Work in being undertaken to develop a licence for its use in different settings… We have gifted the Northland version including a version in Māori for a Māori language emersion high school to the health providers there. (Youth CHAT; Good-year Smith, F, personal communication, Nov 2022).  e.g. ‘Researchers, practitioners, policy makers, and AI/AN-serving organizations may use this example to improve the relevance, efficacy, and use of other mHealth interventions to reach high-risk, underserved populations. BRAVE lessons can be easily integrated into the flow of services provided by clinics, schools, treatment centers, and other community-based programs and can be tailored to the needs and time constraints of any setting [35]. Future research may address the recommendations offered by study participants to improve campaigns for future users and assess the uptake of the service outside the study setting. Native youth and young adults can now text BRAVE to 97779 or STEM to 97779 to receive the SMS text message sequence on their own.’ (BRAVE; Rushing 2021)(2)  e.g. ‘A useful next step would be to consider linking the BRAVE intervention to school-, clinic-, or community-based mental health programs for AI/AN youth as a way to enhance the intervention. Doing so could help build connections to local health services and destigmatize help-seeking, an important goal of the BRAVE SMS series.’ (BRAVE; Wrobel, 2022) (13) |

References:

1. Litwin L, Hankey J, Lucassen M, Shepherd M, Singoorie C, Bohr Y. Reflections on SPARX, a self-administered e-intervention for depression, for Inuit youth in Nunavut. Journal of Rural Mental Health. 2023;47(1):41-50.

2. Rushing SC, Kelley A, Hafner S, Stephens D, Singer M, Bingham D, et al. The BRAVE study: Formative research to design a multimedia intervention for american indian and alaska native young adults. American Indian & Alaska Native Mental Health Research: The Journal of the National Center. 2021;28(1):71-102.

3. Povey J, Sweet M, Nagel T, Lowell A, Shand F, Vigona J, et al. Determining Priorities in the Aboriginal and Islander Mental Health Initiative for Youth App Second Phase Participatory Design Project: Qualitative Study and Narrative Literature Review. JMIR formative research. 2022;6(2):e28342.

4. Povey J, Sweet M, Nagel T, Mills PPJR, Stassi CP, Puruntatameri AMA, et al. Drafting the Aboriginal and Islander Mental Health Initiative for Youth (AIMhi-Y) App: Results of a formative mixed methods study. Internet Interventions. 2020;21:100318.

5. Shepherd M, Fleming T, Lucassen M, Stasiak K, Lambie I, Merry SN. The Design and Relevance of a Computerized Gamified Depression Therapy Program for Indigenous Maori Adolescents. Journal of Medical Internet Research. 2015;17(3):1-.

6. Tighe J, Shand F, McKay K, Mcalister T-J, Mackinnon A, Christensen H. Usage and Acceptability of the iBobbly App: Pilot Trial for Suicide Prevention in Aboriginal and Torres Strait Islander Youth. JMIR Ment Health. 2020;7(12):e14296.

7. Fleming TM, Dixon RS, Merry SN. 'It's mean!' The views of young people alienated from mainstream education on depression, help seeking and computerised therapy. Advances in Mental Health. 2012;10(2):195-203.

8. Fletcher R, Hammond C, Faulkner D, Turner N, Shipley L, Read D, et al. Stayin' on Track: the feasibility of developing Internet and mobile phone-based resources to support young Aboriginal fathers. Aust J Prim Health. 2017;23(4):329-34.

9. Dingwall KM PJ, Sweet M, Friel J, Shand F, Titov N, Wormer J, Mirza T, Nagel T. The Aboriginal and Islander Mental Health Initiative for Youth (AIMhi-Y) app: A non-randomized pilot with First Nations young people. JMIR Preprints.06/06/2022:40111.

10. Black Dog Institute. The extension of iBobbly: an app to reduce suicidality among young Aboriginal and Torres Strait Islander people. New South Wales, Australia: Black Dog Institute; 2015.

11. Wexler L, Gubrian A, Griffin M, DiFulvio G. Promoting Positive Youth Development and Highlighting Reasons for Living in Northwest Alaska Through Digital Storytelling. Health Promotion Practice. 2013;14.

12. Bohr Y LL, Hankey j, McCauge H, Singoorie C, Lucassen M, Shepherd S, Barnhardt J. Evaluating the Utility of a Psychoeducational Serious Game in Protecting Inuit Youth from Depression: A SPARX Pilot Modified Randomized Control Study. JMIR Preprints. 2023;0504/2022:38493.

13. Wrobel J, Silvasstar J, Peterson R, Sumbundu K, Kelley A, Stephens D, et al. Text Messaging Intervention for Mental Wellness in American Indian and Alaska Native Teens and Young Adults (BRAVE Study): Analysis of User Engagement Patterns. JMIR formative research. 2022;6(2):e32138.
